# A Hybrid Method for Mobile Agent Moving Trajectory Scheduling using ACO and PSO in WSNs

**DOI:** 10.3390/s19030575

**Published:** 2019-01-30

**Authors:** Yu Gao, Jin Wang, Wenbing Wu, Arun Kumar Sangaiah, Se-Jung Lim

**Affiliations:** 1 College of Information Engineering, Yangzhou University, Yangzhou 225000, China; gaoyuyz@163.com (Y.G.); jinwang@csust.edu.cn (J.W.); 2 Hunan Provincial Key Laboratory of Intelligent Processing of Big Data on Transportation, School of Computer & Communication Engineering, Changsha University of Science & Technology, Changsha 410000, China; wu_wenbing@163.com; 3 School of Information Science and Engineering, Fujian University of Technology, Fuzhou 350000, China; 4 School of Computing Science and Engineering, Vellore Institute of Technology (VIT), Vellore 632014, India; arunkumarsangaiah@gmail.com; 5 Department of Computer Engineering, Chonnam National University, Yeosu 596597, Korea

**Keywords:** wireless sensor network, mobile agent, ant colony optimization, particle swarm optimization, moving trajectory

## Abstract

Wireless Sensor Networks (WSNs) are usually troubled with constrained energy and complicated network topology which can be mitigated by introducing a mobile agent node. Due to the numerous nodes present especially in large scale networks, it is time-consuming for the collector to traverse all nodes, and significant latency exists within the network. Therefore, the moving path of the collector should be well scheduled to achieve a shorter length for efficient data gathering. Much attention has been paid to mobile agent moving trajectory panning, but the result has limitations in terms of energy consumption and network latency. In this paper, we adopt a hybrid method called HM-ACOPSO which combines ant colony optimization (ACO) and particle swarm optimization (PSO) to schedule an efficient moving path for the mobile agent. In HM-ACOPSO, the sensor field is divided into clusters, and the mobile agent traverses the cluster heads (CHs) in a sequence ordered by ACO. The anchor node of each CHs is selected in the range of communication by the mobile agent using PSO based on the traverse sequence. The communication range adjusts dynamically, and the anchor nodes merge in a duplicated covering area for further performance improvement. Numerous simulation results prove that the presented method outperforms some similar works in terms of energy consumption and data gathering efficiency.

## 1. Introduction

The rapid development of tiny electronic sensors has accelerated the widespread use of wireless sensor networks (WSNs) [1,2,3]. WSNs are usually composed of numerous sensors deployed randomly to detect a target area for some physical features such as temperature, humidity, and gas concentration, and it has been widely applied in smart home [4,5], smart health [6], environment detecting [7], and certain intelligent systems technologies [8,9,10,11]. These sensors are battery-powered and are self-organized in order to construct wireless sensor networks. Traditional WSNs usually adopt static sinks, and that may cause hot spot problems, which makes nodes close to the sink experience premature death due to the heavy burden of data forwarding. The hot spot problems can be mitigated using special clustering and well-designed topology controls, but they cannot solve the issue fundamentally. Recent research on sink mobility technology provides a series of novel ideas to address the problem of hot spots and has achieved great results [12,13,14,15,16,17,18,19,20,21,22,23,24,25,26,27,28,29].

One of the problems demanding prompt solution for sink mobility is the trajectory scheduling of the mobile agent. An efficient traveling path of the mobile agent can reduce the energy consumption and mitigate network latency as well. However, the path scheduling problem has been proven to be a non-deterministic polynomial hard problem (NP-hard) [12,13]. Therefore, it is difficult to acquire the optimal solution to the problem, and many researchers regard it as traveling salesman problem (TSP) [14]. Some intelligent heuristic methods such as ant colony optimization (ACO) [30], particle swarm optimization (PSO) [31], and glowworm swarm optimization (GSO) [32] are introduced for mobile agent path planning and they achieve some results. Many algorithms aim to search for a shorter path for the mobile agent and this inevitably increases the energy consumption during transmission. Therefore, it is hard to make a trade-off between different properties, and the routing protocol designing should be combined with specific applications. Another great achievement is the technology of MU-MIMO, which permits multiple receivers and senders to communicate at the same time by equipping multiple antennas [33,34,35,36]. A mobile agent with MU-MIMO can gather data more efficiently when it accesses more than one target.

The main contribution of this work can be concluded as follows. Firstly, we partition the target area into subdomains using virtual grids, and the cluster heads (CHs) are elected according to their weights. Secondly, the ACO algorithm is executed by the sink node to calculate an optimal sequence of CHs for traversing. Thirdly, the PSO algorithm is executed to select anchor nodes for mobile agents based on the traverse sequence obtained by the ACO algorithm. Fourthly, the communication range of the CHs are adjusted, and some anchor nodes can be merged to balance inter-cluster energy and to economize the sojourn time of the mobile agent. Finally, numerous simulations prove that the presented algorithm HM-ACOPSO plans a more efficient traveling path for the mobile sink as compared to some similar works. Section 2 introduces some of the latest work related to the enhancement of the performance of the network utilizing ACO or PSO and sink mobility technology. Section 3 describes the model of the network and energy and makes some basic assumptions. The traditional ACO and PSO algorithms are explained in Section 4. Section 5 details our presented HM-ACOPSO, and Section 6 demonstrates the simulation parameters of this work. Section 7 shows the simulation results and includes some analyses. Section 8 presents some discussions and proposes some future work. Section 9 concludes the paper.

## 2. Related Work

In this section, we make a brief summary of recent research which focuses on routing protocols designing and utilizing mobile technology and the ACO or PSO algorithm in WSN. A brief summarization of these protocols is shown as Table 1.

### 2.1. Mobile Agent-Based Routing Protocols

In Reference [15], the authors discuss the data gathering problem in order to maximize the volume of collected information in networks with self-charging sensors. By means of convex optimization, they present a distributed data collecting schema to approximate the optimal solution. In Reference [16], the authors adopt two mobile agents to gather information collaboratively. The moving trajectory of mobile agents is characterized by a Fermat spiral route, and it can be predicted according to the constant velocity and the direction of the mobile agents. The sojourn times of the clusters are different for further energy balancing.

### 2.2. ACO-Based Routing Protocols

In Reference [17], the authors present an energy-efficient coverage optimization algorithm called ACB-SA. ACB-SA introduces ant colony optimization (ACO) to address the network coverage problem. In ACB-SA, each sensor can switch between the status of active and inactive freely, and the target of this work is to adopt as few active sensors as possible in order to maintain optimal network coverage area. 

In Reference [18], the authors present an energy-efficient routing schema called ACO-MNCC. It utilizes the ACO algorithm to search the maximum connected covers that disjoint with each other to enhance the lifetime of the network. The pheromone denotes the experiences of hunting, and the heuristic information represents the attraction of the target sensor. Numerous experiments illustrate the energy efficiency of the presented algorithm, especially in heterogeneous WSNs. In Reference [19], the authors present a novel sensor deployment method called ACO-MCC3D in 3-D space coordinates. ACO-MCC3D contains mainly two parts. In phase-I, an improved ACO algorithm is implemented to find the potential location for node deployment, and the heuristic denotes the number of the target points the potential sensor covers. In phase-II, the redundant potential sensors will be deleted. 

An improved version of ACO which utilizes three different pheromones is presented in Reference [20]. One of them used local pheromones to minimize the number of sensors and maintain a certain coverage rate. The other two pheromones are global pheromones used for active sensors selection. In Reference [21], the authors use ACO to avoid the communication blind district and decrease the cost of deployment. A load-balanced deployment schema is designed to improve the connectivity of the network. In Reference [22], the ACO algorithm is utilized for mobile sink moving trajectory scheduling. Nodes in the same subdomain select a CH in accordance with their residual energy, and the traveling path of the mobile sink is transformed into a TSP problem to minimize the network latency. The mobile sink needs to walk to each CH for data gathering.

Authors in Reference [23] present an energy-aware routing method called LTAWSN. Not only the transmission path but also the residual energy among the path is considered to update the pheromone by implementing the ACO algorithm. Simulation results prove that the selected routing path is more reasonable and energy-balancing. Authors in Reference [24] present a hybrid method which combines fuzzy logic, unequal clustering, and ACO technology called FUCHAR. During the clustering phase, sensors are divided into clusters with different sizes in accordance with their residual energy, distance from the sink, neighbor distance, degree of nodes, and so on. The network topology is mainly constructed by means of ACO to hunt for an optimal path. Additionally, the clusters are marked with different levels according to their distance from the sink, and cross-level data forwarding is conducted when necessary. Authors in Reference [25] present an original routing schema called CRT2FLACO. In CRT2FLACO, the type-2 fuzzy logic is utilized for better clustering by considering the features of residual energy, neighbor nodes amount, and destination distance. Then, ants link all the CHs for inter-cluster transmission. 

### 2.3. PSO-Based Routing Protocols

In Reference [26], the PSO algorithm is utilized for cluster forming and inter-cluster routing. Two different kinds of sensors are randomly deployed in the target area. Nodes with higher initial energy not only detect the information of surroundings but also function as gateways for data forwarding. A novel method adopted PSO is presented to determine which gateway should be chosen as the forwarder. In Reference [27], authors enhance the performance of the algorithm presented in Reference [26] and pay more attention to energy balancing. A special particle-encoding method and multi-target fitness method are created to achieve a balance between energy efficiency and energy balancing.

In Reference [28], a novel idea for a clustering-adopting PSO has been presented in EPMS. The authors utilize several straight lines to partition the whole sensor area into subdomains, and each domain denotes a cluster. Each line is represented as an included angle and intercepts with the coordinate axis, and the fitness function describes the quality of the clustering. In Reference [29], the authors present a variable dimension PSO method for mobile sink moving trajectory planning. The mobile sink stays at the rendezvous points for data collection and the number of rendezvous is not determinate. The authors also present a novel method for particle updating using the nearest dimension. 

Protocols such as ACB-SA, ACO-MCC3D, and TPACO, which do not introduce clustering technology, are restricted to small scale networks because the sink node has no assistants and it needs to process all packages. Meanwhile, protocols which only adopt a static sink like ACB-SA, ACO-MNCC, and DSBA may cause hot spot problems and result in nodes’ premature death. One of the big challenges this field faces is how to combine clustering with sink mobility technology to enhance the performance of the network. Additionally, all the protocols in Table 1 are centralized, which means nodes in those protocols need control messages to have guidance for their next action. Distributed protocols are demanded to decrease the burden of control messages. 

## 3. System Model

### 3.1. Basic Assumptions

The following are some assumptions made to simplify the network for the purpose of conducting the experiments conveniently.

- All the sensors maintain static after deployment and they can adjust their communication distance within the communication range.
- All the sensors have the same initial energy and they are all battery-powered. Once the sensor exhausts its energy, it will be useless.
- We assume that the wireless communication link is ideal and there is no collision during data transmission and receiving.
- The mobile agent is carried by an intelligent vehicle and it does not have a limitation of energy. The MU-MIMO technology is introduced for one-to-many communication. The speed and the direction of the mobile agent can be controlled freely.
- The computation process is conducted in a sink node and the residual energy of sensors can be predicted by computation and verified in the transmitted data.

### 3.2. The Network Model

**Figure 1:**
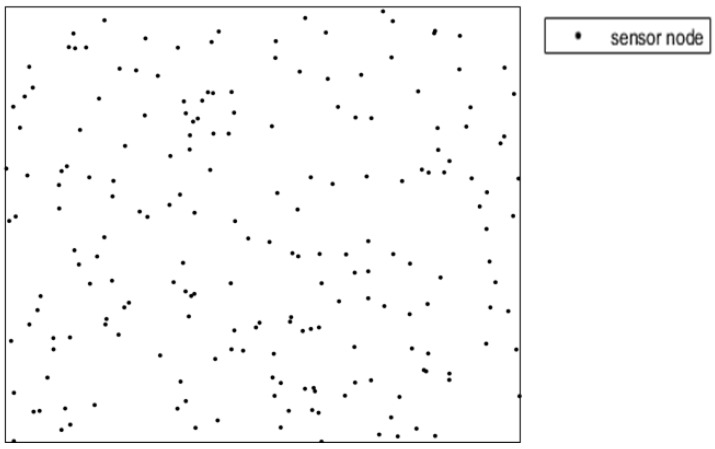
The network model.

In this paper, the sensor field is a rectangular area, and numerous sensor nodes are randomly deployed by plane or other tools, as shown in Figure 1. Each sensor owns its unique ID which is used to recognize it. Sensor data is generated according to time, and sensors have memory for data storage.

### 3.3. The Energy Model

**Figure 2:**
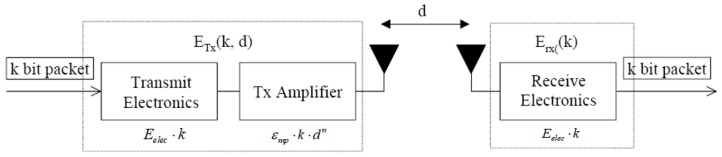
The energy model.

As the literature records [37,38], the energy consumption of sensors used in transmission is about 1000 times higher than the energy used for other purposes. Therefore, we only consider energy consumption used for transmission in our model, which is shown as Figure 2. 

As Figure 2 describes, the energy used in transmission is generally composed of two parts, the sending and receiving parts. The energy used in sending depends on the transmission distance. Once the signal is sent by the transmitter, it will be strengthened by the amplifier, and the amplifier will choose two different power levels to strengthen the signal according to the transmission distance. Therefore, the energy model used in sending data is also classified into two different types, the free space model ($d^{2}$ power loss) and multi-path fading model ($d^{4}$ power loss). The energy used for sending the *l*-bit data package can be calculated as:

(1) $$E_{Tx}(l,d)=\left\{ \begin{array}{ll} l\cdot E_{elec}+l\cdot \varepsilon_{fs}\cdot d^{2} & if,d<d_{0}\\ l\cdot E_{elec}+l\cdot \varepsilon_{mp}\cdot d^{4} & if,d\geq d_{0} \end{array}\right.$$

where $E_{elec}$ is the energy use of the transmitter and receiver unit. $\varepsilon_{fs}$ and $\varepsilon_{mp}$ denote the amplification coefficients for two different models, respectively. $d_{0}$ is the distance threshold for choosing different amplifier powers, which can be calculated as:

(2) $$d_{0}=\sqrt{\frac{\varepsilon_{fs}}{\varepsilon_{mp}}}$$

The energy used for the receiver is simpler compared to the sending part, and its power is constant, the same as the transmitter. The energy used for receiving the *l*-bit data package can be calculated as:

(3) $$E_{Rx}=l\cdot E_{elec}$$

## 4. Overview of Traditional ACO and PSO Algorithms

**Figure 3:**
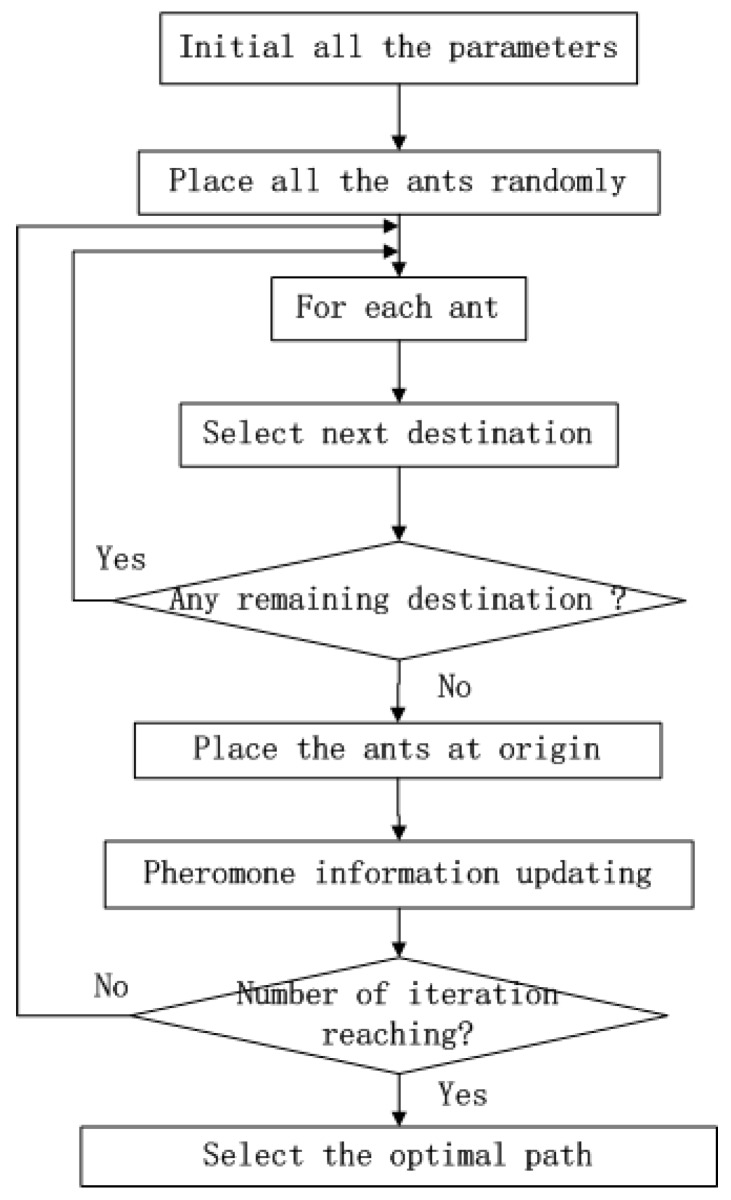
The workflow of ACO.

Ant colony optimization (ACO) is a searching method determined by means of previous experience [30] and enlightened by the food-hunting process of ants. When ants walk through a path, they release a material called pheromone, and pheromone can volatilize after a while. In the beginning, ants choose each path with equal probability, and the path with the shorter length costs less time for traveling. Therefore, the path with shorter length will be travelled by more ants in a given time, and the pheromone in those paths become higher. In cycles, more ants will choose the short path for food hunting to reduce the total time cost. ACO is applied to address the traveling salesman problem (TSP) [14,39] originally and it has been widely used in various fields. The working procedure of ACO is described in Figure 3.

**Figure 4:**
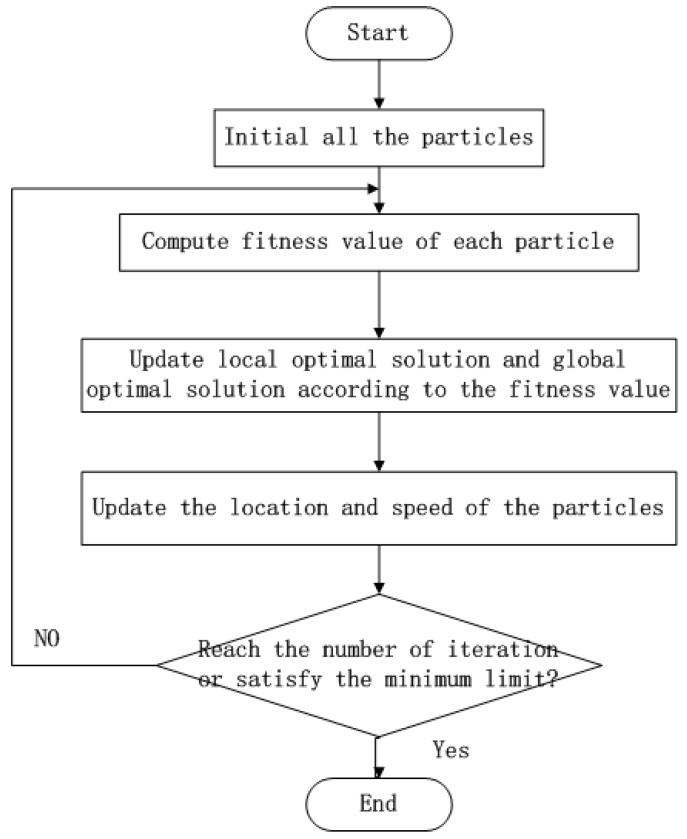
The workflow of PSO.

PSO is another intelligent heuristic algorithm which utilizes the wisdom of the swarm [31,40,41]. Each particle commonly represents a whole solution for the problem, and the fitness function evaluates the performance of the solution represented by the particle. During each iteration, a local and a globally-optimal solution are marked for updating the location of the particles. Particles search for better solutions according to local and global optimal solutions. The working procedure of PSO is described as in Figure 4. 

## 5. Our Presented HM-ACOPSO Algorithm

In this section, we will illustrate our presented HM-ACOPSO algorithm. HM-ACOPSO mainly contains four phases: Clustering; mobile agent shortest moving sequence searching based on ACO; further improvement of the scheduled path using anchor nodes based on PSO; and communication range adaption for energy balancing and anchor nodes merging for decreasing the sojourn time of the mobile agent. During the initial phase of the network, sensors exchange their own information that includes location and sensor ID with their neighbors in the range of communication. 

### 5.1. Sub-Domain Division

We utilize a virtual grid to divide the sensor field into subdomains, and each subdomain represents a cluster. In order to simplify the intra-cluster communication, cluster members only communicate with their corresponding cluster head directly. In order to guarantee that any two nodes in the same subdomain can communicate with each other, the length of each subdomain should satisfy the following formula:

(4) $$w=\frac{\sqrt{2}}{2}R$$

where R is the communication range of ordinary sensors. Each subdomain elects a cluster head in accordance with the weight of each node, and the weight can be calculated using Formulas (5)–(7):

(5) $$W_{i}=\frac{E_{residual}}{D_{total}}$$

(6) $$D_{total}=\sum_{i\in A}dis{\left(i,j\right)}^{2}$$

(7) $$dis(i,j)=\sqrt{{\left(x_{i}-x_{j}\right)}^{2}+{\left(y_{i}-y_{j}\right)}^{2}}$$

where $E_{residual}$ is the residual energy of node i and A is the set of neighbors of node i. 

**Figure 5:**
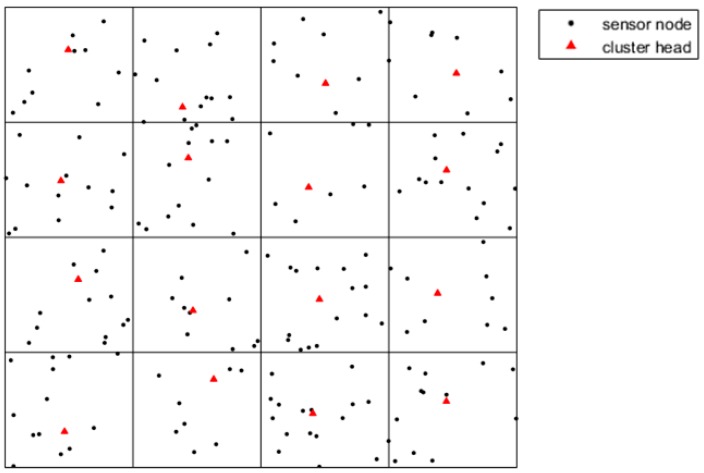
The sensor field after clustering.

During the initial phase of election, nodes close to the subdomain center take the lead in broadcasting a competition package which contains the node ID and weight. Neighboring nodes compare their own weights with the receiving package. If the neighbor nodes own the bigger weight, they will broadcast their own competition package. Finally, nodes with the biggest weight will be chosen as CH. The process of clustering needs to create many control messages which increase the extra energy consumption. Therefore, the network conducts the clustering procedure after several rounds. Figure 5 describes the result of the clustering.

### 5.2. Shortest Path Selection Based on ACO

For some delay-tolerant applications such as environment monitoring, there is no need for multi-hop communication among cluster heads which will result in a heavy forwarding burden on CHs. In our proposed HM-ACOPSO algorithm, we use a mobile agent for data collection. During each round, the mobile agent needs to traverse all the CHs and sojourn for data gathering. The moving trajectory of the mobile agent has a significant impact on the performance of the network. A vital feature for moving trajectory planning is the length of the path. A short moving path will cost less time for the mobile agent traveling. In this section, we utilize the ACO algorithm for shortest path selection. We use an undirected complete graph G=<V,E> to represent the network and V denotes the set of cluster heads and E denotes the set of edges between any two cluster heads. The following steps are used to conduct the ACO algorithm.

**Step 1:**m ants are randomly placed in n CHs and a matrix with m×n dimension is used to record each ant’s traveling path.

**Step 2:** The possibility of next cluster heads that ants will choose can be calculated using Formula (8):

(8) $$p_{ij}^{k}(t)=\left\{ \begin{array}{ll} \frac{\tau_{ij}^{\alpha }(t)\cdot \mu_{ij}^{\beta }(t)}{\sum_{k\in \mathrm{next}}\tau_{ik}^{\alpha }(t)\cdot \mu_{ik}^{\beta }(t)} & \mathrm{if}\;j\in \mathrm{next}\\ 0 & \;\mathrm{otherwise} \end{array}\right.$$

where $p_{ij}^{k}$ denotes the possibility that the kth ant travels from CH i to CH j and next denotes the set of CHs that ant k has not travelled. τ is the pheromone, and μ is the inspired factor, which is the reciprocal of the distance between CH i to CH j. Each ant travels all the CHs in accordance with the above possibility. α and β are two constant control factors.

**Step 3:** A fitness function is utilized to evaluate the quality of the traveling path of the ants’ and it can be defined as follows:

(9) $$Fitness(k)=\sum_{e\in Path}e$$

where Path denotes the set of edges the ant travels. The best travel path is recorded as the parameters for PSO.

**Step 4**: The pheromone will volatilize after a while and when an ant travels through a path, it will emit a pheromone. Therefore, the pheromone can be updated as:

(10) $$\tau_{ij}(t+1)=(1-\rho )*\tau_{ij}(t)+\Delta \tau_{ij}$$

(11) $$\Delta \tau_{ij}=\sum_{k=1}^{m}\Delta \tau_{ij}^{k}$$

(12) $$\Delta \tau_{ij}^{k}=\left\{ \begin{array}{ll} \frac{Q}{fitness(k)} & \mathrm{if}\;\mathrm{ant}-k\;\mathrm{pass}\;{\mathrm{path}}_{ij}\\ 0 & \mathrm{otherwise} \end{array}\right.$$

where ρ is the evaporation rate.

**Figure 6:**
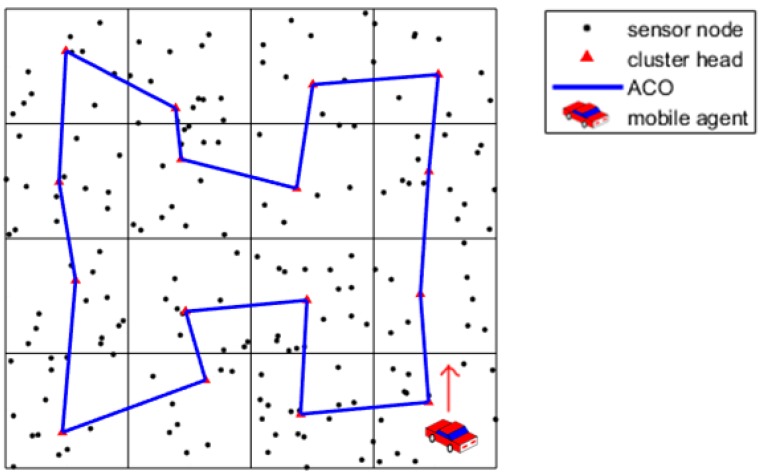
Shortest path selection using the ACO algorithm.

**Step 5**: Repeat steps 1–4 until reaching the necessary number of iterations. Finally, we record the path with the shortest length. The sample after path selection using the ACO algorithm is shown as Figure 6.

The pseudocode of the ACO-based shortest path selection is described in Algorithm 1.

**Algorithm 1**: Pseudocode of ACO-based shortest path selection1: **Input:** Set of CHs *C =* {$C_{1},C_{2}\ldots C_{n}$}, distance between $CH_{i}$ and $CH_{j}$$C_{ij}$, pheromone $Tabu_{ij}$, set of visited CHs *C_Visit*, the probability of next CH for visiting P2: **Output:** Shortest Path *L =* {$A_{1},A_{2}\ldots A_{n}$}3: **Step 1:**4: Initialize *m* ants, $C_{ij}$ and $Tabu_{ij}$5: **Step 2:**6: **for**
*i* = 1 to *Iteration Number*
**do**7:  Reinitialize *C_Visit*8:  Randomly place *m* ants in *n* CHs9:  **for**
*j* =1 to *m*
**do**10:   **for**
*k*=1 to *n*
**do**11:    Calculate P /* Using formula (8) */12:    Select next visit $CH_{k}$ according P13:    Add $CH_{k}$ into *C_visit*14:   **end for**15:  **end for**16:  Calculate Fitness(m) /* Using formula (9) */17:  Record the best path *L*18:  Update $Tabu_{ij}$ /* Using formula (10), (11) and (12)*/19: **end for**20: Return the best path *L*21: **Step 3: Stop**

### 5.3. Anchor Nodes Selection Based on PSO 

As mentioned above, the mobile agent needs to move to the location of the CHs for data collecting. However, each sensor owns its fixed wireless communication coverage, and any two sensors which are in each other’s communication range can communicate with each other freely. Therefore, the mobile agent has no occasion to reach CHs for data collecting, and the shortest path selected by ACO can be further optimized. We allocate a virtual anchor node for each cluster head and we utilize the PSO algorithm for anchor nodes selection. We still use the order that the preceding ACO achieves to traverse the anchor nodes of the CHs. Each particle represents a path across all the anchors, and we assume that the current order for traversing is $A=\{a_{1},a_{2},a_{3}\cdots \cdots a_{n}\}$. The dimension of each particle is 2n and the particle swarm can be denoted as:

(13) $$P=\left[\begin{array}{l} p^{1}\\ p^{2}\\ p^{3}\\ \;\vdots \\ p^{n} \end{array}\right]=\left[\begin{array}{c} x_{a_{1}}^{1},y_{a_{1}}^{1},x_{a_{2}}^{1},y_{a_{2}}^{1}\cdots \cdots x_{a_{n}}^{1},y_{a_{n}}^{1}\\ x_{a_{1}}^{2},y_{a_{1}}^{2},x_{a_{2}}^{2},y_{a_{2}}^{2}\cdots \cdots x_{a_{n}}^{2},y_{a_{n}}^{2}\\ x_{a_{1}}^{3},y_{a_{1}}^{3},x_{a_{2}}^{3},y_{a_{2}}^{3}\cdots \cdots x_{a_{n}}^{3},y_{a_{n}}^{3}\\ \vdots \\ x_{a_{1}}^{n},y_{a_{1}}^{n},x_{a_{2}}^{n},y_{a_{2}}^{n}\cdots \cdots x_{a_{n}}^{n},y_{a_{n}}^{n} \end{array}\right]$$

where $x_{a_{i}}^{k},y_{a_{i}}^{k}$ denotes the location of kth anchor. The target of the PSO algorithm is to minimize the traveling path of the mobile agent and we still adopt the fitness function as the ACO used. We set an initial speed for each dimension of the particle and the restriction of speed and location of particles are as follows:

(14) $$limit(v_{i}^{k})=[-10,10]$$

(15) $$dis(anchor^{k},CH^{k})<R$$

We use the following steps to execute our POS algorithm.

**Step 1**: We randomly set the initial location and speed of particles. The location and speed satisfy the restriction of Formulas (14) and (15).

**Step 2:** The fitness value of particles is calculated in accordance with Formula (9). Each particle compares the current fitness value with its previous optimal fitness value and chooses the better one as its $P_{best}$. Similarly, we compare the current global fitness value with the previous optimal global fitness value and choose the better one as $G_{best}$.

**Step 3**: The speed and the location of particle $p^{i}$ is updated using the following formulas respectively.

(16) $$V_{i}(t+1)=\eta V_{i}(t)+c_{1}\times rand()\times (P_{ibest}-P_{i}(t))+c_{2}\times rand()\times (G_{best}-P_{i}(t))$$

(17) $$P_{i}(t+1)=P_{i}(t)+V_{i}(t+1)$$

where η denotes the inertia factor. $c_{1}$,$c_{2}$ denote the weight factor and they satisfy $c_{1}+c_{2}=1$.

**Step 4:** After updating the speed and location, each particle checks whether the value of its speed and location exceed the boundary. If the speed exceeds the limit, it will be set as the boundary value, and if the location exceeds the limit, the location information will not be updated.

**Step 5**: Next, it conducts step 2 and iterates until reaching the maximal iteration number.

**Figure 7:**
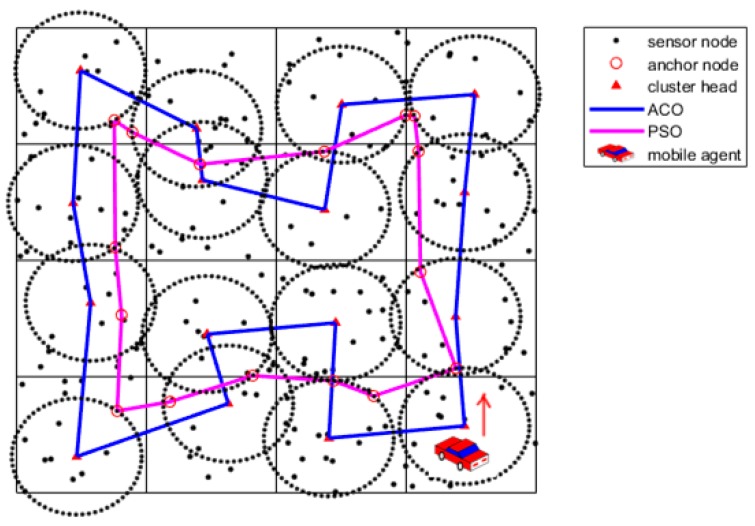
Anchor nodes selection using the PSO algorithm.

Finally, $G_{best}$ represents the solution for the anchor nodes selection. The sample after the anchor nodes selection is shown as Figure 7.

The pseudocode of the PSO-based anchor nodes selection is described in Algorithm 2.

**Algorithm 2**: Pseudocode of PSO-based anchor nodes selection1: **Input:** Location of CHs *Loc_CH*, particle matrix *P*, velocity of particle *V*, the shortest path *L* from Algorithm 12: **Output:** Optimal anchor nodes *Anchor =* {$a_{1},a_{2}\ldots a_{n}$}3: **Step 1:**4: Initialize particle matrix *P* according to *Loc_CH*, *L* and formula (15), randomly initialize *V* according to the formula (14)5: **Step 2:**6: **for** i = *1* to *Iteration Number*
**do**7:  Calculate Fitness(particle) /* Using formula (9) */8:  **for**
*j* = 1 to *n*
**do**9:   $P_{jbest}$ = {$P_{jbest}$|Fitness($Particle_{j}$))} 10:  **end for**11:  $G_{best}$ = {$G_{best}$|min(Fitness(*Particles*))}12:  **for**
*j* = 1 to *n*
**do**13:   Update velocity $V_{j}$ of $Particle_{j}$ /* Using formula (16) */14:   Update *P* /* Using formula (17) */15:   Check the boundary of $Particle_{j}$16:  **end for**17: **end for**18: Return the optimal anchor nodes *Anchor =* {$a_{1},a_{2}\ldots a_{n}$}19: **Step 3:** Stop

### 5.4. Communication Range Adjustment

**Figure 8:**
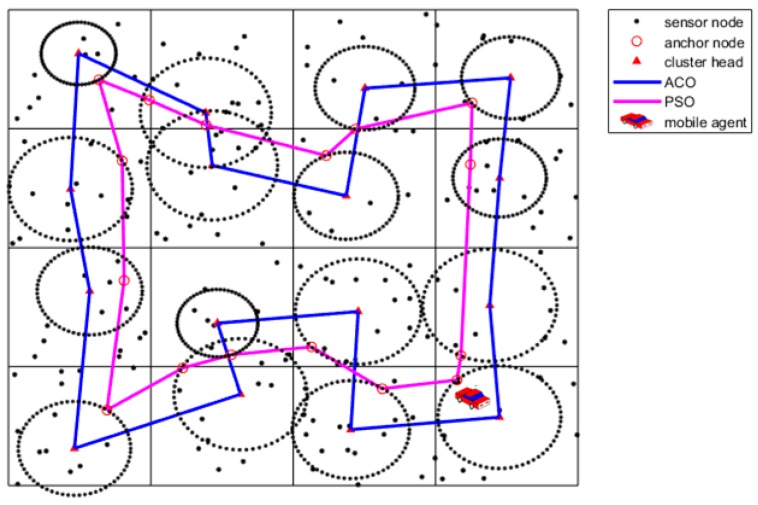
Communication range adjustments.

During the initial round, sensors own the same initial energy, and the network is energy-balanced. As the number of rounds increases, the network becomes energy-heterogeneous due to the uneven deployment and heavy burden of forwarding. The clustering addresses the problem of energy unbalancing within the cluster, whereas, the unbalanced energy of the inter-cluster is still unsolved. In this paper, we adopt the method of sensor communication range adjustment to handle the unbalanced energy between different clusters. The CHs commonly consumes the most energy during the transmission with the mobile agent, and we only consider adjusting the communication range of the CHs. The communication range of each CHs is dynamic and is changed according to Formula (18).

(18) $$R_{CH_{i}}=(1-c\frac{E_{Max}-E_{CH_{i}}+\varepsilon }{E_{Max}-E_{Min}+\varepsilon })R$$

where $E_{Max}$ denotes the maximal residual energy of CHs and $E_{Min}$ denotes the minimal residual energy of CHs. c is a regulatory factor between 0 and 1, and it usually is set as 0.5. ε is a very small constant to avoid the denominator to be zero. The sample after communication range adjustment is shown as Figure 8.

### 5.5. Anchor Nodes Merging

The total traveling time contains two parts, including moving time and sojourn time. The total time the mobile agent uses to complete a whole travel path can be calculated using the following formula.

(19) $$T_{total}=\frac{L_{total}}{v}+N_{anchor}\cdot t_{sojourn}$$

The sojourn time is related to the number of anchor nodes. More anchor nodes mean that the mobile agents have to spend more time data collecting. In this paper, the MU-MIMO technology is adopted to enable the mobile agent to collect the data from multiple sensor nodes. If any anchor node is in the transmission range of multiple CHs simultaneously, we can merge the relevant anchor nodes. Each anchor node is checked to determine whether it is in multiple CHs’ transmission ranges utilizing the following equation:

(20) $$\sqrt{{\left(x_{i}-x_{△}\right)}^{2}+(y_{i}-y_{△})}\leq R\&\&\sqrt{{\left(x_{j}-x_{△}\right)}^{2}+(y_{j}-y_{△})}\leq R$$

where $\left(x_{i},y_{i}\right)$,$\left(x_{j},y_{j}\right)$ are the coordinates of any two CHs and $\left(x_{△},y_{△}\right)$ is the coordinate of any anchor node. We only reserve the anchor nodes which are in a conjunct communication range and remove the other anchor nodes.

## 6. Simulation Environment

### 6.1. Network Parameters

With the purpose of having an evaluation of our proposed HM-ACOPSO algorithm, we employ Matlab to simulate the experiment. We also compare some similar works such as ACO-TSP, VD-PSO, and LEACH with this work to highlight the outstanding performance of this work. The relevant parameters used in this paper are listed in Table 2.

### 6.2. ACO Parameters

In the ACO algorithm, the parameters α, β and ρ have great influence on the performance of the ACO. We make numerous attempts to find a better combination of the parameters to enhance the performance of ACO. Numerous simulations are shown in Table 3, and from Table 3, we can see that the best values for α, β and ρ are 1, 2, and 0.5 respectively.

### 6.3. PSO Parameters

In the PSO algorithm, η, $c_{1}$, and $c_{2}$ also have a significant effect on the final anchor nodes selection and iteration. Different combinations of these parameters and their performances are described in Table 4. From Table 4, we can see that the best values for η, $c_{1}$, and $c_{2}$ are 0.8, 0.4, 0.6, respectively.

## 7. Performance Evaluation

**Figure 9:**
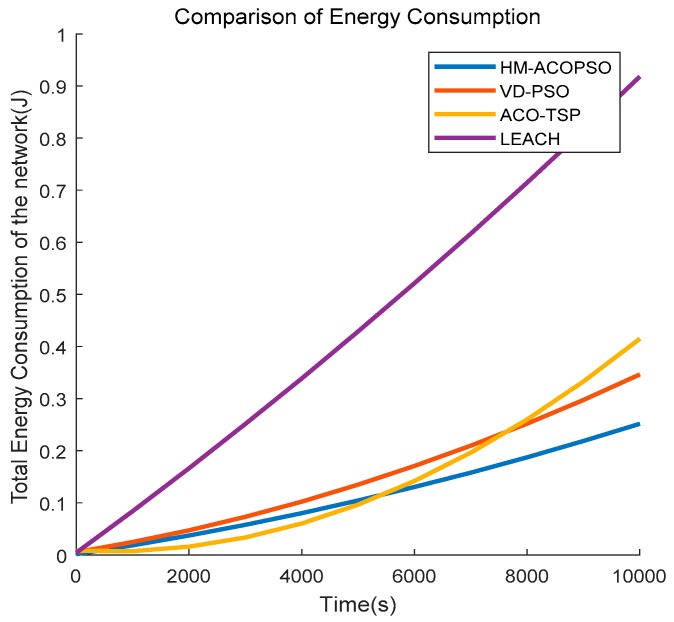
Total energy consumption of the network.

We first analyze the energy consumption of the network between different algorithms. As illustrated in Figure 9, as time rises, the energy consumption of the four algorithms increases, and LEACH rises more rapidly. We know that each of the four algorithms adopts a clustering algorithm. However, CHs are more unevenly distributed in LEACH. Some nodes may be far away from the CHs and that results in long-distance communication which consumes significantly more energy. HM-ACOPSO, VD-PSO, and ACO-TSP all introduce mobile data collectors for data gathering which conserve energy. The energy consumption of ACO-TSP increases most slowly before 5500 s because the mobile agent in ACO-TSP needs to access each CH and the transmission distance between the mobile agent and CHs is very close. Whereas, the CHs selection in ACO-TSP only considers the residual energy which causes significant energy dissipation in intra-cluster communication. VD-PSO takes energy balances into consideration so that it schedules a moving trajectory with much more energy consumption, but the energy of the whole network is much more balanced. Therefore, the energy increases steadily in VD-PSO. Our proposed HM-ACOPSO method features a more reasonable clustering method and the scheduled path is optimized so that it achieves better performance in aspects of energy consumption.

**Figure 10:**
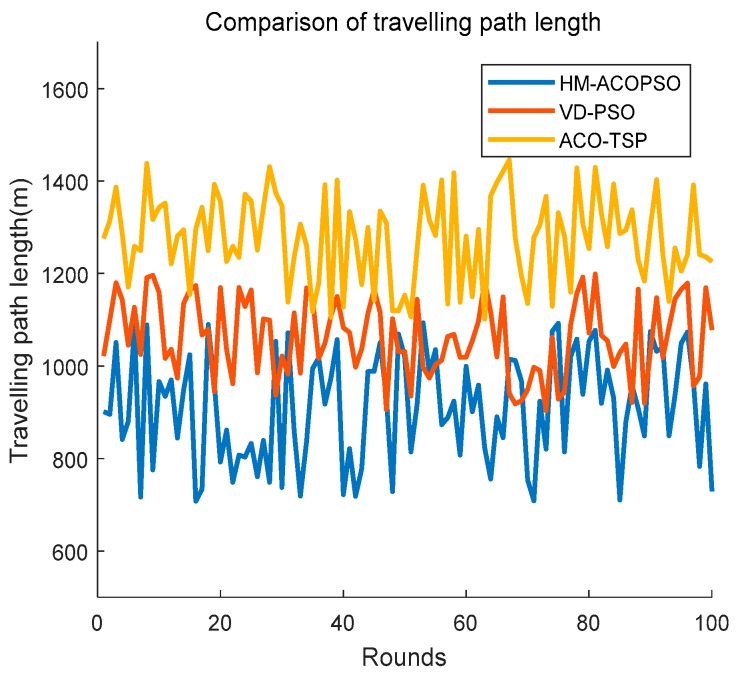
The traveling path length of the mobile agent.

We also research the traveling path that different algorithms schedule, and the result is described as Figure 10. We can clearly see from Figure 10 that HM-ACOPSO almost has the best performance in terms of scheduled path length. There is no doubt that the ACO-TSP achieves the worst performance in terms of traveling path length because the mobile agent in ACO-TSP needs to access each CH closely to collect information. VD-PSO schedules rendezvous points of mobile agents to sojourn for information gathering, and that is much more efficient compared to ACO-TSP. However, VD-PSO does not make full use of wireless communication range and it sacrifices some performance for balancing the energy of the whole network. One of the great improvements in HM-ACOPSO is that it makes full use of the wireless communication range, and therefore, it can schedule the optimal traveling path for the mobile agent.

**Figure 11:**
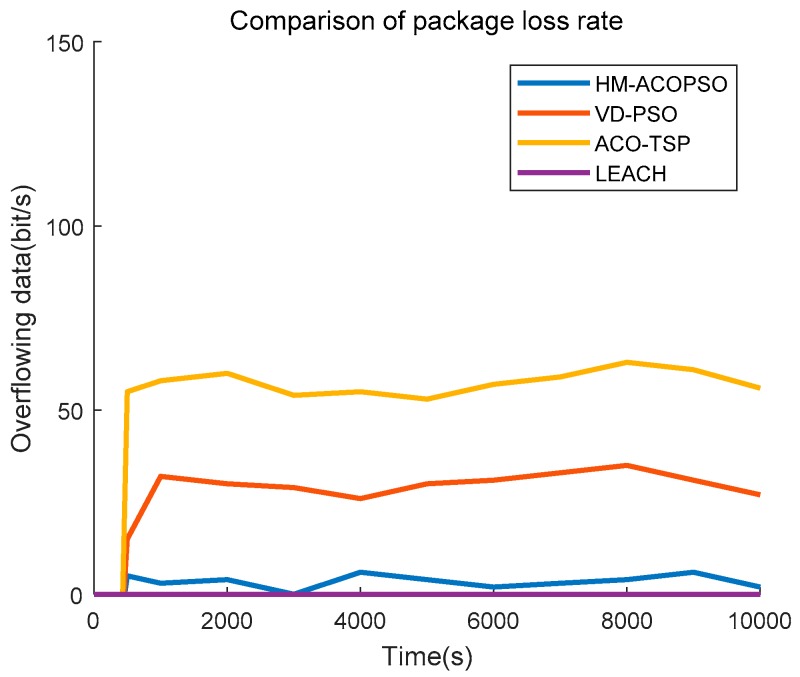
Overflowing data of the network.

We then study the package loss rate of each algorithm. We assume that all of the communication is successful and that the packages will be dropped only when the memory of the sensors overflows. The result is demonstrated as Figure 11. The package loss rate has great relevance to the efficiency of the mobile agent data gathering. In LEACH, the sink node is static, and all the data is transmitted to it timely by multiple-hop communication. Therefore, package loss will not occur. Whereas, in HM-ACOPSO, VD-PSO, and ACO-TSP, the mobile agent is introduced to collect data, and the shorter the traveling path and the less the sojourn points are, the more efficient the mobile agent is. Our proposed HM-ACOPSO method makes full use of the wireless communication range to shorten the mobile agent traveling path and merge the sojourn points to enhance the work efficiency of the mobile agent. A long traveling path of a mobile agent is likely to induce a high package loss rate.

**Figure 12:**
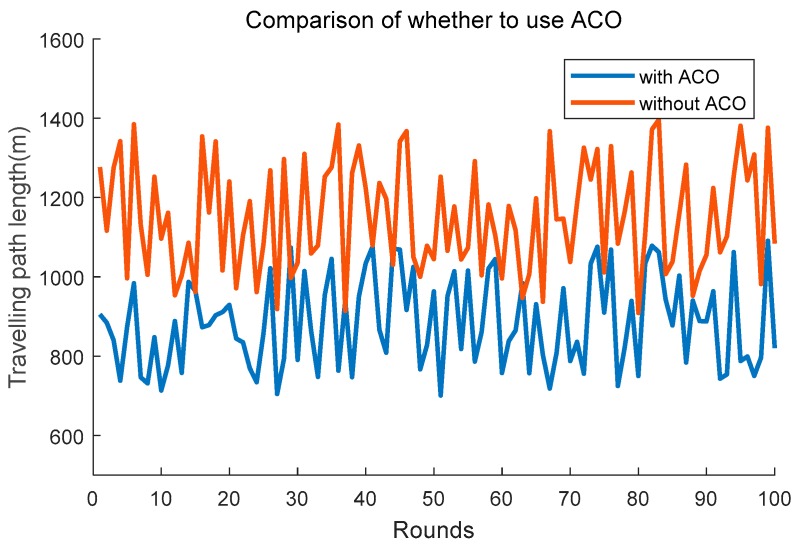
Comparison of whether to adopt ACO.

The input of PSO needs to use the output of ACO. Specifically, when we construct the particles *P* which represent anchor nodes, we need to use the sequence the ACO created to order the CHs. We also compare it with the anchor nodes ordered in a random way. The simulation result is shown as Figure 12. We can clearly see that the schema without ACO performs worse than the schema using ACO. The upper limit of the schema which doesn’t adopts ACO is close to twice that of the schema which adopts ACO. Even if the sets of anchor nodes in the two schemas are the same, when the mobile agent travels them in a different order, the result could be very different. That is also the reason why we need to combine the ACO with PSO rather than only using PSO algorithm. 

## 8. Discussion and Future Work

Sink mobility as an emerging technology is widely used in various types of WSNs. One important issue which needs to be addressed is moving path scheduling which has a significant effect on the performance of the mobile agent. Much research has been done to explore efficient data gathering methods for mobile agents, and the moving schema is generally divided into three types, including the controlled moving schema, the uncontrolled moving schema, and the random moving schema. Different moving schema are applied to different applications. In the controlled moving schema, we hope the traveling path is as short as possible to decrease the network latency and reduce the loss package rate. Meanwhile, the shortest traveling path means that the mobile agent may not approach nodes and cause extra energy consumption. Meanwhile, the predictability of the mobile agent greatly enhances the reliability of the network because nodes can have the exact location of the mobile agent and the routing can be scheduled in advance. The schema of the uncontrolled moving and random moving are mainly used in applications such as endangered animal detecting. The mobile agent is usually placed on the animals and moves with the animals, which may result in network isolation.

The traveling path planning should be determined by the specific application which demands low energy consumption or low network latency and package loss rate. In our proposed HM-ACOPSO algorithm, we also take energy balancing into consideration. The communication range of each CHs can be adjusted dynamically according to their residual energy so that the weak CHs will be protected using a close communication distance. Additionally, more mobile agents can also be adopted to collect data cooperatively to reduce energy consumption and network latency at the same time, if the budget allows for this.

Our future work will mainly focus on multiple mobile agents cooperative work which can further enhance the performance of the network. Multiple mobile agents cooperative work faces challenges as follows: Firstly, mobile agents need to communicate with each other to collect data cooperatively. How can the mobile agents communicate with each other efficiently? Then we must determine how to schedule the traveling path of each agent, which is much harder than for multiple agents than for a single mobile agent network. Finally, as the mobile agent number increases, the control messages among the network increase exponentially. How can we decrease the control messages to simply the network? These problems must be solved.

## 9. Conclusions

In this paper, we presented an efficient moving path scheduling method called HM-ACOPSO. We firstly partition the whole sensor field into several subdomains utilizing virtual grids, and then the CHs are selected in accordance with their weights. An efficient traveling loop is planned using a hybrid method with ACO and PSO. The communication range of CHs can be adjusted according to residual energy to protect the weak nodes, and anchor nodes can be merged to save sojourn time. Finally, the mobile agent moves along a predefined trajectory to traverse all the sojourn points for data collection. The simulation results prove that our presented method outperforms similar methods in terms of energy consumption and package loss rate. 

## Figures and Tables

**Table 1:** Routing protocols which adopt ant colony optimization (ACO) and particle swarm optimization (PSO) algorithms.

Protocol Name	Year	Targets	Methods	Sink Type	Clustering	Topology Control	Network Size
ACB-SA [17]	2012	Energy efficient, coverage optimizing	ACO based	single static sink	False	Centralized	Small
ACO-MNCC [18]	2012	Network lifetime enhancing	ACO based	multiple static sinks	False	Centralized	Middle
ACO-MCC3D [19]	2018	Deployment optimization	ACO based	single static sink	False	Centralized	Small
TPACO [20]	2011	Network lifetime enhancing	ACO based	single static sink	False	Centralized	Small
DSBA [21]	2015	Deployment optimization, blindness avoiding	ACO based	single static sink	False	Centralized	Small
ACO-TSP [22]	2016	Mobile sink shortest moving path scheduling	ACO based	single mobile sink	True	Centralized	Large
LTAWSN [23]	2015	Network lifetime enhancing	ACO based	single static sink	False	Centralized	Middle
FUCHAR [24]	2018	Network lifetime enhancing	ACO, fuzzy logic, unequal clustering	single static sink	True	Centralized	Large
CRT2FLACO [25]	2015	Load balancing, network lifetime enhancing	ACO, type-2 fuzzy logic, clustering,	single static sink	True	Centralized	Large
PSO [26]	2014	Energy efficient	PSO, clustering	single mobile sink	True	Centralized	Large
PSO-IMPROVE [27]	2016	Energy efficient and energy balancing	PSO, clustering	single mobile sink	True	Centralized	Large
EPMS [28]	2016	Network lifetime enhancing	PSO, clustering	single mobile sink	True	Centralized	Large
VD-PSO [29]	2016	Mobile sink moving path scheduling	PSO, clustering	single mobile sink	True	Centralized	Large

**Table 2:** Network parameters.

Parameter Name	Parameter Value
Network size	400 × 400 m
Number of sensors (N)	200
Memory capacity of sensors	500 bits
Communication range of sensors (R)	[0,50] m
Data generation ratio of sensors (l)	1 bits/s
Initial energy of sensors ($E_{0}$)	0.05 J
Mobile agent moving speed (v)	2 m/s
Mobile agent sojourn time ($E_{0}$)	5 s
Energy consumption on circuit ($E_{elec}$)	50 nJ/bit
Free-space channel parameter ($\varepsilon_{fs}$)	10 pJ/bit/m^2^
Multi-path channel parameter ($\varepsilon_{mp}$)	0.0013 pJ/bit/m^4^

**Table 3:** Optimal path length under different ACO parameter combinations.

α	β	ρ	Optimal Path Length	Iteration Number
0.5	0.5	0.5	2031.6	65
0.5	1	0.5	1737.4	62
1	1	0.5	1548.7	58
1	2	0.5	1548.7	45
1	3	0.5	1548.7	54
2	1	0.5	1588.5	55
1	2	0.4	1548.7	48
1	2	0.6	1548.7	60

**Table 4:** Optimal path length under different PSO parameters combination.

η	$c_{1}$	$c_{2}$	Optimal Path Length	Iteration Number
0.8	0.5	0.5	1015.9	33
0.8	0.4	0.6	983.6	29
0.8	0.3	0.7	1024.6	32
0.8	0.6	0.4	1005.6	34
0.7	0.4	0.6	1064.4	38
0.9	0.4	0.6	1026.1	34

## Data Availability

The data that support the findings of this study are available from the corresponding author upon reasonable request.
